# Is Joint Denervation a Durable Treatment for Thumb Carpometacarpal Osteoarthritis? A Multicenter Long-Term Outcome Study

**DOI:** 10.1097/PRS.0000000000012905

**Published:** 2026-08-26

**Authors:** Niek J. Nieuwdorp, Camille Blaaker, Esmee Kwee, Erik T. Walbeehm, Ruud W. Selles, Kyle R. Eberlin, J. Michiel Zuidam, Hand-Wrist Study Group, R. Arjen M. Blomme, Caroline A. Hundepool

**Affiliations:** Rotterdam, the Netherlands; and Boston, MA; From the 1 Department of Plastic, Reconstructive, and Hand Surgery; 5 Department of Rehabilitation Medicine, Erasmus MC; 2 Hand and Arm Center, Department of Orthopaedic Surgery; 3 Division of Plastic and Reconstructive Surgery, Department of General Surgery, Massachusetts General Hospital, Harvard Medical School; 4 Hand and Wrist Center, Xpert Clinics.

## Abstract

**Background:**

Joint denervation for thumb carpometacarpal (CMC) osteoarthritis (OA) remains less commonly used, primarily due to concerns about the long-term durability of symptom relief. This study aims to assess the long-term reoperations and outcomes after CMC denervation and to identify prognostic factors associated with postoperative pain.

**Methods:**

This multicenter prospective study included patients with thumb CMC OA who underwent joint denervation. The primary outcome was the rate of reoperation due to recurrent CMC joint symptoms. Secondary outcomes included long-term pain and hand function measured using the visual analog scale (score, 0 to 100), as well as patient satisfaction.

**Results:**

A total of 69 patients (76 thumbs) undergoing CMC denervation were included. In 30% of cases, an additional partial wrist denervation (posterior interosseous nerve and anterior interosseous nerve) was performed for CMC OA. After a median follow-up of 30 months (range, 6 to 110 mo), 28% of thumbs underwent reoperation for recurrent complaints, with a median time to reoperation of 9 months. Visual analog scale pain and function scores improved significantly by 3 months and remained stable at long-term follow-up among those not requiring reoperation. At long-term follow-up, 66% of patients without reoperation reported that they would choose to undergo denervation again. Additional partial wrist denervation (*P* = 0.001) and scaphotrapeziotrapezoid involvement (*P* = 0.042) were associated with less pain improvement, whereas male sex was associated with more improvement (*P* = 0.023).

**Conclusions:**

Joint denervation is an effective and durable treatment for CMC OA, with 72% not undergoing reoperation and reporting sustained improvements in pain and function. Additional partial wrist denervation did not show superior results and is therefore not recommended.

Current treatment strategies for thumb carpometacarpal (CMC) osteoarthritis (OA) range from nonsurgical approaches, including hand therapy, splinting, and intra-articular corticosteroid injections, to surgical interventions when symptoms become refractory.^1^ Currently, partial or complete trapeziectomy with or without ligament reconstruction has been the principal surgical approach. In recent years, however, joint prosthesis has gained traction as a promising alternative.^2,3^ Yet, for all their benefits, these procedures carry a risk of complications arising from their invasive nature.^4–10^ Furthermore, these invasive surgical procedures require prolonged immobilization and extended recovery times, delaying return to work and daily activities.^11,12^

In recent years, CMC joint denervation has attracted growing attention as a less invasive alternative,^13^ offering the potential for significant pain reduction without compromising joint integrity. By only severing the small sensory nerve branches to the joint, denervation allows for a short postoperative immobilization period with no expected loss of strength or mobility and, therefore, allows a faster return to function.^14–16^ Moreover, it has been associated with a lower risk of complications compared with more invasive surgical options.^17–19^

Despite the potential advantages of CMC joint denervation, the procedure is not widely performed.^13^ Many surgeons remain hesitant due to limited and low-quality evidence, particularly concerning its long-term effectiveness.^20–22^ The lack of evidence leaves a gap in understanding the durability of symptom relief and its effectiveness in preventing invasive reoperations. Therefore, this study aims to assess the incidence of reoperations during long-term follow-up after thumb CMC joint denervation. Secondary objectives include long-term pain and hand function, as well as treatment satisfaction, complications, and return to work. In addition, we explore the factors associated with reoperation and postoperative pain outcomes.

## PATIENTS AND METHODS

### Study Design and Setting

In this multicenter, prospective cohort study, data were collected as part of routine outcome measurements at multiple specialized hand surgery and therapy clinics. Ethical approval was obtained from the medical research ethical committee of the Erasmus MC, and all patients provided informed consent. Outcomes up to 12 months were routinely collected at fixed timepoints during clinical care.^23^ For this study, patients who were at least 18 months post denervation were invited to complete an additional questionnaire. This study was conducted following the Strengthening the Reporting of Observational Studies in Epidemiology guidelines.^24^

### Participants

This study included patients with thumb CMC OA who underwent surgical joint denervation between March of 2016 and November of 2024. The diagnosis was established by a Federation of European Societies for Surgery of the Hand–certified hand surgeon based on clinical evaluation and physical examination, including the grind and crank test.^25^ Radiographic imaging was performed to confirm chondropathy of the CMC or scaphotrapeziotrapezoid (STT) joint.^26^ Initial management consisted of hand therapy and/or an orthosis provided by a certified hand therapist. Surgical intervention was considered in cases where nonoperative treatment failed to provide adequate symptom relief.

Patients were excluded if they presented with thumb CMC instability,^27,28^ had a history of CMC joint surgery, or had a history of preceding trauma. Eligibility criteria were assessed using medical records and the data collection system.

### Surgical Technique

**Fig. 1.**
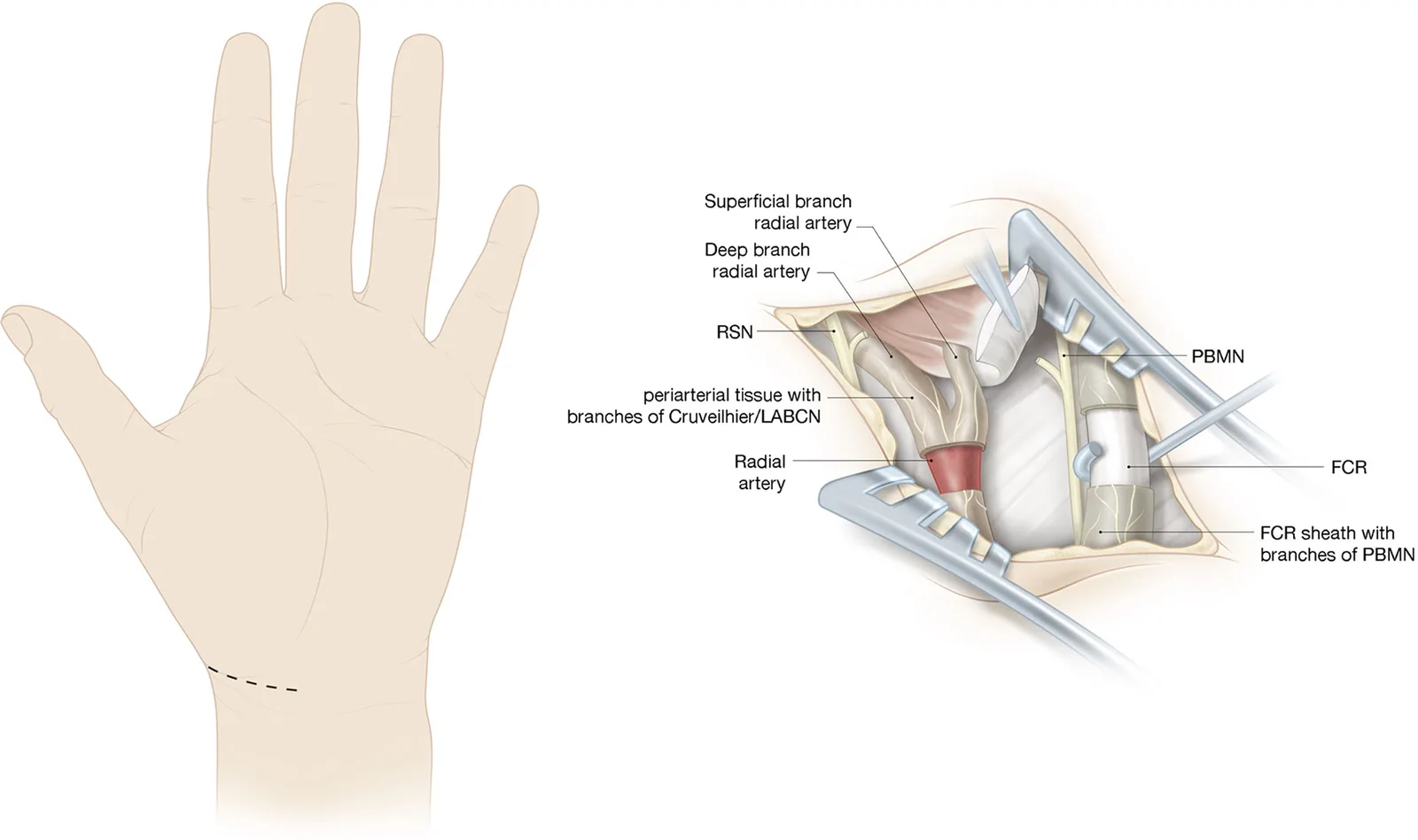
Thumb CMC joint denervation through a volar approach. A proximally extending Wagner incision provides access for selective denervation of articular branches, including the radial sensory nerve (*RSN*), lateral antebrachial cutaneous nerve (*LABCN*), and palmar cutaneous branch of the median nerve (*PBMN*), located around the sheath of the flexor carpi radialis (*FCR*) tendon. Reprinted with permission from Tieman TE, Duraku LS, van der Oest MJW, Hundepool CA, Selles RW, Zuidam JM. Denervation of the joints of the hand and wrist: surgical techniques and a systematic review with meta-analysis. *Plast Reconstr Surg.* 2021;148:959e–972e.

The denervations were performed by 7 Federation of European Societies for Surgery of the Hand–certified surgeons with level 3 to 5 expertise,^29^ under general or regional (supraclavicular or axillary) anesthesia. A proximally extending Wagner incision was made, preserving cutaneous branches of the radial sensory nerve (Fig. 1). A dorsoradial dissection plane was created from the base of the first metacarpal toward the second metacarpal and distal radius, transecting the radial sensory nerve branches to the joint capsule. Beneath the first extensor compartment, branches of the lateral antebrachial cutaneous nerve were identified and transected. The articular branches of the lateral antebrachial cutaneous nerve were transected through careful dissection around a 1-cm segment of the radial artery. Next, the proximal thenar musculature was partially detached from the base of the first metacarpal to expose and transect articular branches of the palmar branch of the median nerve. Additional distal palmar branches of the median nerve were transected by mobilizing the soft-tissue layer over the scaphoid tubercle toward the flexor carpi radialis tendon. Distal articular branches of the anterior interosseous nerve (AIN) could optionally be transected through the same incision. Diathermic ligation of the CMC joint capsule was performed to complete the denervation. The thenar musculature was repositioned and secured, followed by skin closure and application of a pressure bandage or plaster cast. Slight modifications to this technique were made based on the preference of the surgeon.

**Fig. 2.**
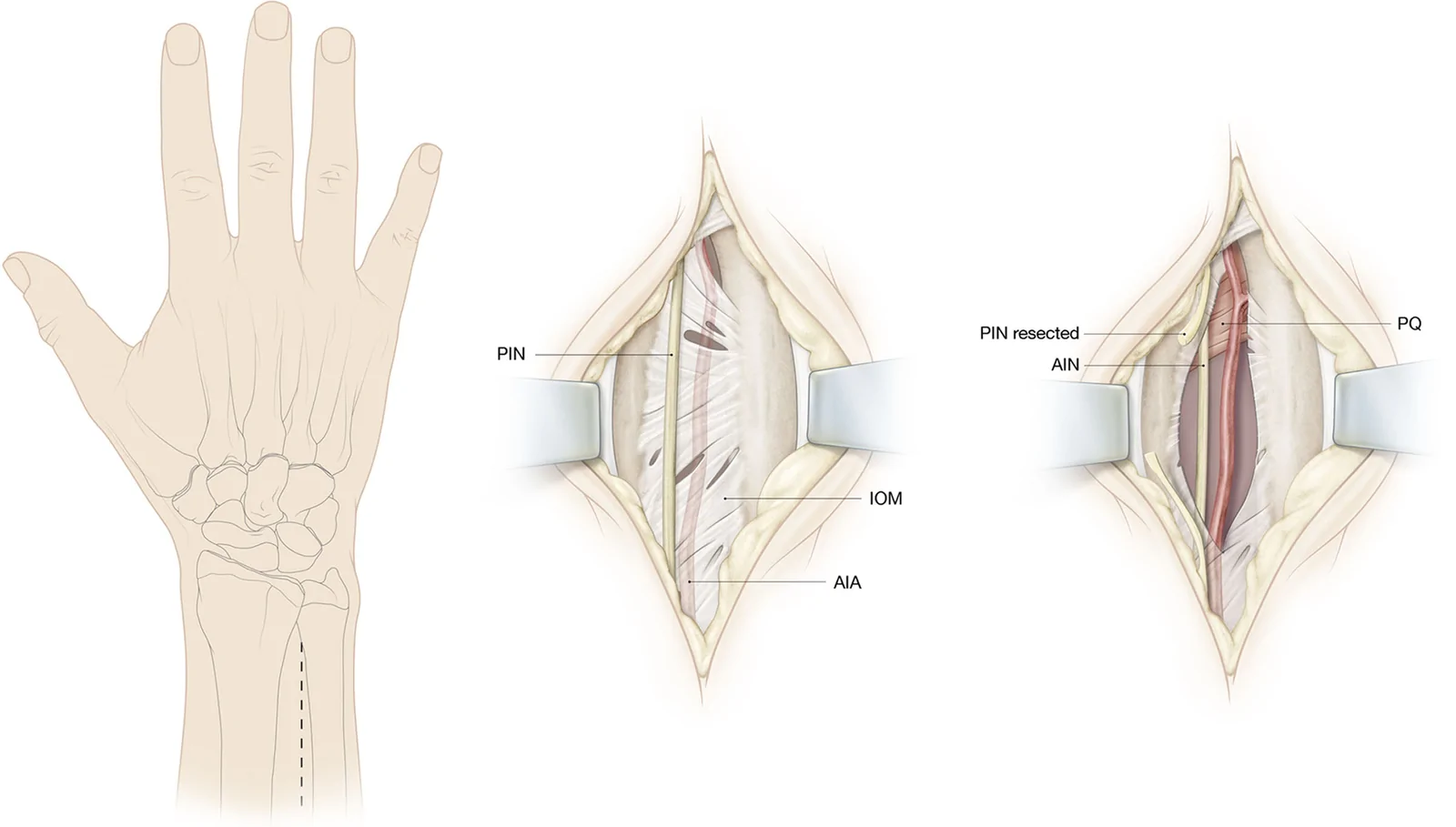
Partial wrist denervation through a dorsal approach. In a subset of patients, this extended denervation was performed as part of thumb CMC joint denervation. A longitudinal dorsal incision provides access for selective denervation of the PIN and AIN, in proximity to the interosseous membrane (IOM), anterior interosseous artery (AIA), and pronator quadratus (PQ) muscle. Reprinted with permission from Tieman TE, Duraku LS, van der Oest MJW, Hundepool CA, Selles RW, Zuidam JM. Denervation of the joints of the hand and wrist: surgical techniques and a systematic review with meta-analysis. *Plast Reconstr Surg.* 2021;148:959e–972e.

In addition, a subset of patients underwent an extended denervation as part of the CMC denervation (Fig. 2),^30^ based on the preference of the surgeon. A dorsal wrist incision was made; the fourth extensor compartment was opened; and the posterior interosseous nerve (PIN) was identified dorsal to the interosseous membrane and resected. The interosseous membrane was subsequently incised to access and resect the AIN on its volar aspect. Wounds were closed in layers, and a pressure bandage or plaster cast was applied.

### Postoperative Rehabilitation

A standardized postoperative rehabilitation protocol was applied according to local guidelines. All patients underwent either pressure bandage or cast-splint immobilization for 3 to 5 days. From that point, patients were allowed to use their hand as tolerated. Patients typically received hand therapy 1 to 2 times per week under the supervision of specialized hand therapists. At 2 weeks postoperatively, functional stability exercises were initiated to improve coordination and restore a stable thumb position. By 7 weeks, rehabilitation shifted to progressively strengthening the thenar muscles and enhancing joint stability. The frequency and duration of therapy varied and were jointly determined by the therapist and the patient.

### Variables and Measurements

The primary outcome of this study was the rate of reoperation for persistent or recurrent thumb CMC joint symptoms after surgical denervation. Data on subsequent reoperations were obtained through review of the medical records and responses of the patients to the follow-up questionnaire.

Secondary outcomes included visual analog scale (VAS) scores (0 to 100) for pain at rest, pain during exertion, and hand function, routinely collected at baseline, 6 weeks, 3 months, and 12 months. Additional long-term data were obtained through a follow-up questionnaire. Higher VAS pain scores indicated greater pain severity, whereas higher VAS function scores reflected better hand function. Patients who underwent CMC joint reoperation during follow-up were excluded from pain and hand function assessments, as the procedure disrupts the evaluation of the original treatment effects. Exclusions were determined through chart reviews and patient-reported data from the long-term questionnaire.

Additional secondary outcomes included patient-reported satisfaction with the treatment outcome, return to work,^11^ and postoperative complications. Patient satisfaction and willingness to undergo the same treatment again were assessed using the Satisfaction with Treatment Result Questionnaire (5-point scale)^31^ until November of 2021, after which the International Consortium for Health Outcomes Measurement questionnaire (7-point scale)^32^ was adopted. For analysis, responses were dichotomized: the top 2 points (5-point scale) and top 3 points (7-point scale) indicated satisfaction. Satisfaction was routinely measured at 3 months, with additional data collected at long-term follow-up. “Return to work” was defined as patients resuming at least half of their usual job duties, based on self-reported questionnaires collected at 6 weeks and at 3, 6, and 12 months after surgery.^11,32^ Complications were classified according to the International Consortium for Health Outcomes Measurement Complications in Hand and Wrist conditions (ICHAW) classification. (**See Table, Supplemental Digital Content 1**, which illustrates the ICHAW tool, https://links.lww.com/PRS/I774.)^10,32,33^

Demographic variables, including sex, age, occupational status, symptom duration, treatment side, and hand dominance, were systematically collected in our database. Involvement of the STT joint, concurrent diagnoses at intake, and additional treatments during follow-up were documented through chart review. The long-term follow-up questionnaire provided supplemental information on treatments received outside our institution.

### Statistical Analysis

For the primary analysis, the reoperation rate after CMC joint denervation was reported using descriptive statistics and visualized using a survival curve. A linear mixed model was used to analyze VAS scores over time, with time as a continuous predictor. All model assumptions were tested and confirmed to be met.

A Cox proportional hazards model was used to explore risk factors of reoperation. To investigate the association of variables with postoperative VAS pain at exertion, we applied a joint model.^34^ This approach accounts for missing pain score data, particularly in patients who underwent a reoperation. Regression coefficients (β) represent the expected change in VAS pain at exertion for a 1-unit increase in the predictor variable, with associated 95% credible intervals (CrIs). Due to the limited number of reoperations, each variable was tested independently in univariable analyses. Variable selection was guided by expert consultation and review of the literature. All model assumptions were verified and met.

Separate nonresponder analyses were conducted to identify significant differences in demographics and preoperative scores between patients who completed the intake, 3-month, or long-term questionnaires (responders) and those who did not (nonresponders). Unpaired *t* tests, Fisher exact tests, chi-square tests, and Wilcoxon rank sum tests were used to compare baseline characteristics. In addition, we used standardized mean differences to quantify imbalance independent of sample size.^35^ The Little test was performed to determine whether data were missing completely at random.^36^

All analyses were performed using R statistical software (version 4.3.0; R Foundation for Statistical Computing, Vienna, Austria). A 95% confidence interval (CI) was used, and *P* values less than 0.05 were considered statistically significant.

## RESULTS

Between March of 2016 and November of 2024, 75 patients (82 thumbs) underwent thumb CMC denervation. After excluding 3 patients with thumb CMC instability, 2 with traumatic OA, and 1 with prior CMC surgery, 69 patients (76 thumbs) were included. The median follow-up was 30 months (range, 6 to 110 mo). The baseline characteristics are presented in Table 1. Included patients were predominantly female (79%), with a mean age of 57 (SD, 10) years. A concurrent surgery was performed in 16% of procedures. (**See Table, Supplemental Digital Content 2**, which provides an overview of concurrent diagnoses and additional surgical procedures performed alongside CMC joint denervation, https://links.lww.com/PRS/I775.) An extended (PIN/AIN) denervation was performed in 30% of cases, with no significant differences in baseline demographics between extended and standard denervation groups. (**See Table, Supplemental Digital Content 3**, which compares the baseline demographic characteristics of patients undergoing standard versus extended denervation, https://links.lww.com/PRS/I776.)

**Table 1.** Demographic Characteristics of the Study Sample at Baseline

Variable	Demographic Characteristics (*N* = 76)
Age, y, mean (SD)	57 (10)
Female sex, *n* (%)	60 (79)
Treated side, *n* (%)	
Left	47 (62)
Right	29 (38)
Dominant side treated, *n* (%)	33 (43)
Occupational intensity, *n* (%)	
Unemployed	18 (24)
Light physical labor (eg, working in an office)	19 (25)
Moderate physical labor (eg, working in a shop)	20 (26)
Heavy physical labor (eg, working in construction)	19 (25)
Second opinion, *n* (%)	9 (12)
Concurrent diagnosis, *n* (%)	21 (28)
STT joint involvement, *n* (%)	16 (21)

*N*, total number of thumbs.

A total of 76 thumbs were eligible for the long-term reoperation rate analysis. Of these, 59 thumbs (77%) had a minimum follow-up of 18 months and were therefore included in the long-term analysis of secondary outcomes. These patients were contacted for follow-up, and 47 patients completed the long-term questionnaire, resulting in a response rate of 80%. The minor differences observed in the nonresponder analysis, together with the nonsignificant Little test (*P* = 0.964), indicate that the data were missing completely at random. (**See Table, Supplemental Digital Content 4**, which illustrates the nonresponder analysis at intake, https://links.lww.com/PRS/I777.) (**See Table, Supplemental Digital Content 5**, which illustrates the nonresponder analysis at 3-month follow-up, https://links.lww.com/PRS/I778.) (**See Table, Supplemental Digital Content 6**, which illustrates the nonresponder analysis at long-term follow-up, https://links.lww.com/PRS/I779.)

### Reoperation Rate

**Fig. 3.**
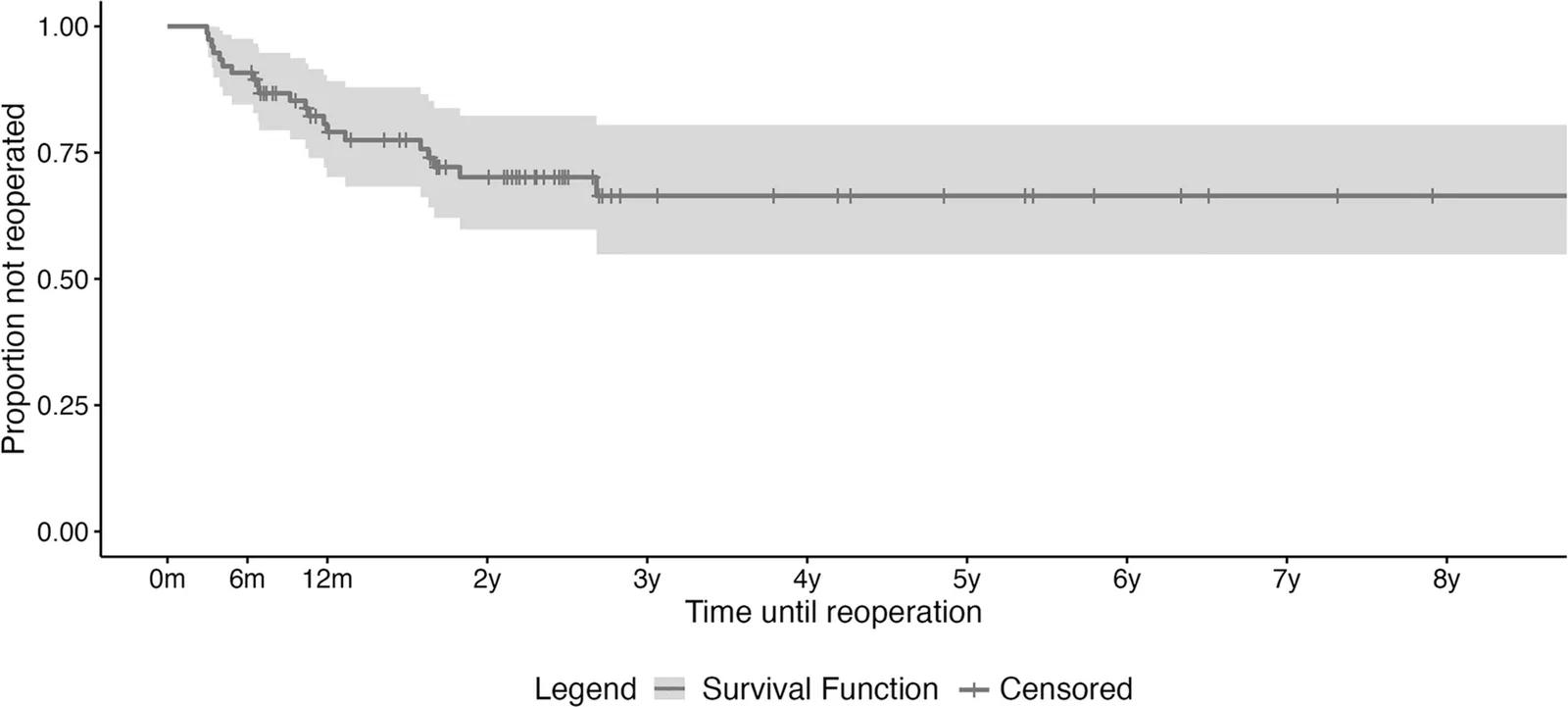
Kaplan–Meier survival curve indicating the proportion of patients who did not undergo reoperation at the CMC joint over time after denervation surgery. At a median follow-up of 30 months, 28% of the thumbs underwent reoperation, with a median time to reoperation of 9 months. The *shaded light gray* area denotes the 95% CI. Censored indicates patients who had not undergone a reoperation at the time of the study.

Figure 3 presents the reoperations over time. At a median follow-up of 30 months, 28% of the thumbs (21 of 76; 95% CI, 18% to 39%) underwent a reoperation due to perceived lack of benefit after denervation. Most of these reoperations (71%) occurred within 12 months after denervation, with a median time to reoperation of 9 months (interquartile range, 4 to 13 mo). Among those who underwent reoperation, 18 received a trapeziectomy, 2 received a CMC prosthesis, and 1 underwent a CMC lipofilling.

### Patient-Reported Pain and Function

**Fig. 4.**
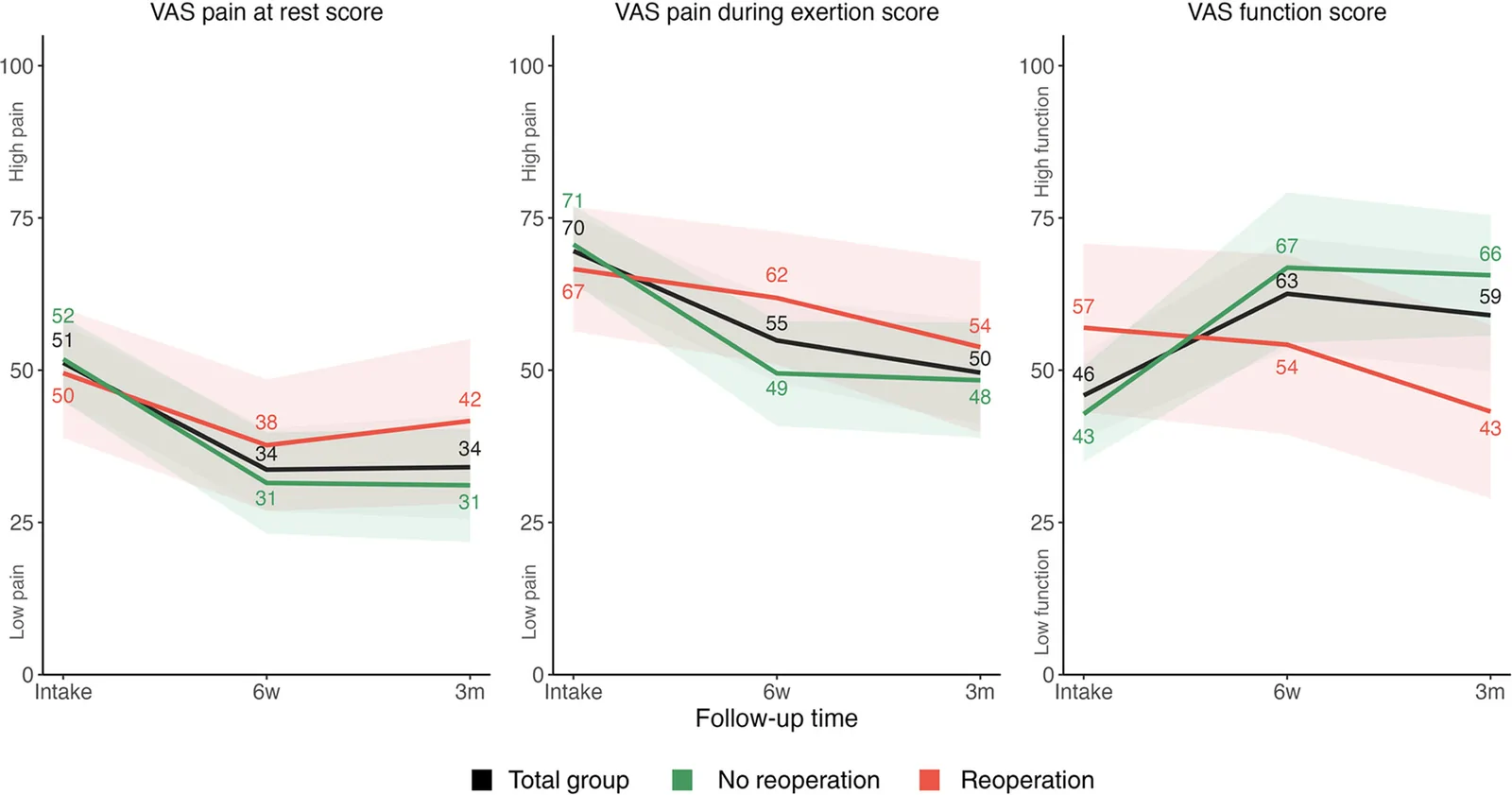
Mean VAS scores measured before denervation, at 6 weeks, and at 3 months after CMC denervation. The *black line* represents the entire patient group, the *red line* indicates patients who eventually underwent reoperation, and the *green line* represents those who did not. *Shaded ribbons* indicate the 95% CIs. A significant improvement in mean VAS scores was observed at 3 months after denervation. Early postoperative outcomes appear to predict the likelihood of reoperation. (*Left*) VAS pain at rest score. (*Center*) VAS pain during exertion score. (*Right*) VAS function score.

**Fig. 5.**
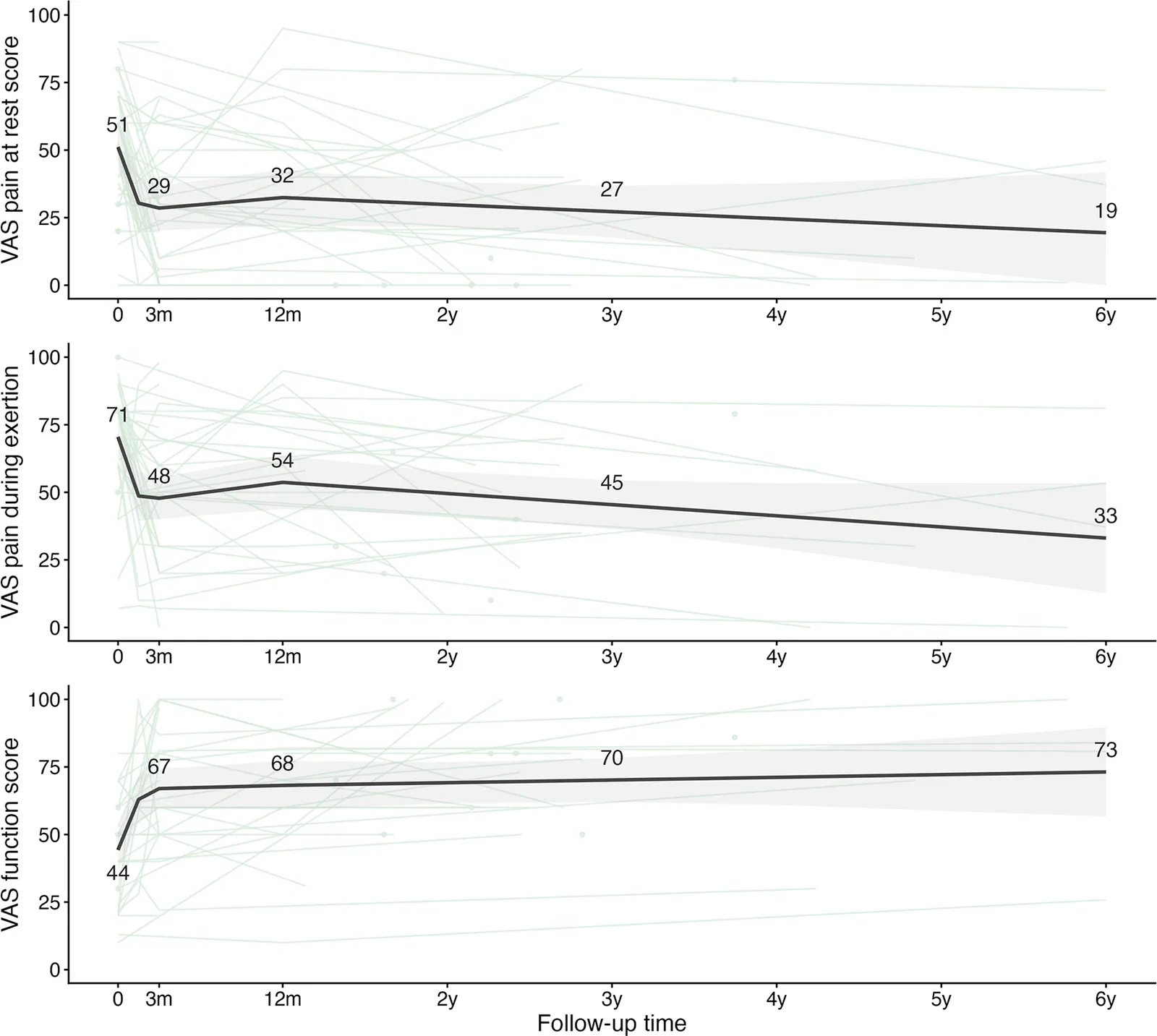
The mean VAS scores before denervation and at 3 months, 12 months, and over the long-term follow-up for the patients who did not undergo CMC joint reoperation during follow-up. The *shaded ribbon* indicates the 95% CI, with individual patient scores plotted in *light green*. The mean VAS scores showed significant improvement at 3 months after denervation and remained stable thereafter. A large between-subject variability was observed. These results represent only patients who did not undergo CMC joint reoperation during the study period. (*Above*) VAS pain at rest score. (*Center*) VAS pain during exertion score. (*Below*) VAS function score.

The mean VAS pain and function scores improved significantly from intake to 3 months (Fig. 4). To evaluate the long-term pain and function outcome, we analyzed only those patients who did not undergo CMC joint reoperation. In this subgroup, VAS pain and function scores remained stable beyond the 3-month follow-up, with no statistically significant changes. At long-term follow-up, the mean VAS pain score at rest was 30 (95% CI, 20 to 40), the mean VAS pain score during exertion was 46 (95% CI, 37 to 56), and the mean VAS function score was 71 (95% CI, 62 to 79). Despite the sustained improvement in scores, substantial intersubject variability was observed (Fig. 5).

### Satisfaction with the Treatment Result

At long-term follow-up, for the patients who did not require reoperation, 52% (15 of 29) were satisfied and 66% (19 of 29) were willing to undergo the treatment again. The most common reason for patients not willing to undergo treatment again was persistent or recurrent symptoms. Notably, many patients who underwent reoperation remained satisfied with the denervation at long-term follow-up and would choose to undergo it again. (**See Figure, Supplemental Digital Content 7**, which illustrates patient satisfaction and willingness to undergo treatment again at 3 mo and long-term follow-up, https://links.lww.com/PRS/I780.)

### Complications and Return to Work

Within the first year, 6 of 76 denervations (8%) had an ICHAW event (complication) (Table 2). These consisted of minor adverse protocol deviations, of which 4 (67%) were classified as ICHAW grade 1 and 2 (33%) as ICHAW grade 2. Return to work outcomes were assessed in 22 patients who were employed at the time of surgery. Within a 12-month follow-up period, 82% of patients returned to work, with a median return time of 3 (95% CI, 1.5 to 5) weeks.

**Table 2.** Overview of the Number of ICHAW Events and the Number of Thumbs Affected, According to the ICHAW Tool,^10,32,33^ during the First Year after CMC Joint Denervation

	No. of Thumbs *n* (%)	Specification
None	70 (92)	
Grade 1	4 (5)	3 sensory disturbances in scar requiring hand therapy or silicone scar sheet, 1 temporary sensory disturbance in the radial nerve
Grade 2	2 (3)	1 wound infection requiring antibiotics, 1 excessive swelling requiring pressure glove and oral steroids
Grade 3	0 (0)	

### Prognostic Factors

Three factors were significantly associated with postoperative VAS pain during exertion (Table 3). First, men reported a significantly greater pain reduction than women after denervation surgery, with an average difference of 0.85 points on the VAS scale per month (β = −0.85; 95% CrI, −1.51 to −0.10; *P* = 0.023). Second, patients with STT involvement with CMC OA showed a significantly smaller reduction in pain. Their VAS scores decreased 0.47 points less per month than the overall reduction (β = 0.47; 95% CrI, 0.01 to 0.92; *P* = 0.042). Third, patients who underwent an extended CMC denervation demonstrated significantly less improvement in pain compared with those who did not, whereas there were no significant differences in preoperative scores between the groups (**see Table, Supplemental Digital Content 3**, https://links.lww.com/PRS/I776). On average, their VAS pain scores decreased 0.56 points less per month than the overall reduction (β = 0.56; 95% CI, 0.14 to 0.98; *P* = 0.001). Outcomes were generally more favorable in patients without extended denervation. (**See Document, Supplemental Digital Content 8**, which describes outcomes for patients who underwent CMC denervation without extended [PIN/AIN] denervation, https://links.lww.com/PRS/I781.) No other variables were significantly associated with changes in pain scores. None of the factors predicted reoperation. (**See Table, Supplemental Digital Content 9**, which illustrates the Cox proportional hazards model for risk factors for reoperation after CMC denervation, https://links.lww.com/PRS/I782.)

**Table 3.** Factors Associated with Changes in Pain^a^

	β	95% CrI	*P*
Age, y	−0.01	−0.03 to 0.01	0.564
Male sex	−0.85	−1.51 to −0.10	0.023^b^
Dominant side treated, yes	0.42	−0.05 to 0.88	0.072
STT joint involvement, yes	0.47	0.01 to 0.92	0.042^b^
Extended (PIN/AIN) denervation, yes^c^	0.56	0.14 to 0.98	0.001^b^
Concurrent surgery, yes^c^	0.49	−0.10 to 1.08	0.099
Employed, yes	0.10	−0.41 to 0.63	0.718

a Factors associated with changes in pain, measured using the VAS (0 to 100) during exertion over time after CMC denervation, are presented as fixed-effects coefficients (β) with corresponding 95% CrIs. Beta coefficients represent the associations between baseline factors and the monthly changes in VAS pain scores during exertion, where higher scores indicate greater pain. Each variable was tested in univariable analyses.

b Statistically significant (*P* < 0.05).

c The factor was tested using a linear mixed model, as the joint model could not adequately estimate the effect. Therefore, the reported values represent 95% CI for this parameter.

## DISCUSSION

The results of this study indicate that the majority of patients experienced sustained improvements in pain and hand function after joint denervation for thumb CMC OA. The procedure appears to be an effective and durable treatment for approximately 3 of 4 patients, as only 28% underwent a reoperation within the mean follow-up of 30 months. In addition, the procedure offers several advantages, including a low complication rate, rapid recovery, and the ability to pursue trapeziectomy or joint replacement if symptoms persist.

Our observed reoperation rate of 28% provides a valuable estimate in the context of the existing literature, where the reoperation rate remains largely uncertain and reports vary from 0% to 60%.^17,30,37–39^ Importantly, among patients who did not undergo reoperation, we observed a significant and sustained reduction in pain as well as an improvement in hand function. Although some residual pain may persist, these findings align with earlier studies.^14,16,40–42^ However, the long-term outcomes regarding pain, function, and reoperation rates remain insufficiently documented in the existing studies. The current literature is limited by small sample sizes, short follow-up duration, or the inclusion of concurrent CMC joint procedures.^14,16,17,30,37–43^ Our results therefore provide a more reliable benchmark for patient counseling and highlight denervation as a durable and minimally invasive option that may avoid the complications of more extensive procedures altogether.

Our study is the first to systematically report long-term satisfaction after CMC denervation in a prospective cohort of this size. Although these rates are lower than those reported in short-term studies (satisfaction, 61% to 92%; willingness, 75% to 88%), they were assessed using different measures than in previous reports, which limits direct comparability.^17,18,30,37–39,44,45^ Patients most frequently cited persistent or recurrent symptoms as reasons for not choosing the procedure again. This likely reflects the recurrence of symptoms over time, consistent with the variability observed in long-term pain outcomes. Interestingly, satisfaction rates were similar among those who underwent reoperation and those who did not, indicating that revision surgery does not necessarily reflect dissatisfaction with the initial denervation.

CMC denervation has been proposed as a potential direct alternative to procedures such as trapeziectomy with ligament reconstruction and tendon interposition (LRTI).^14–16^ When compared with long-term outcomes of LRTI, pain relief after denervation appears less favorable. Daams et al. ^46^ reported outcomes in 1369 patients undergoing LRTI, showing a decrease in average VAS pain scores from 63 at baseline to 28 at 3 months, and 24 at 4 years postoperatively. The less favorable mean pain relief after CMC denervation may be influenced by substantial variability in outcomes within the group, as illustrated in Figure 5. Although some patients experienced excellent long-term results, others reported limited or no benefit. Despite the lesser symptom relief, CMC denervation offers notable advantages, including faster recovery and a lower complication rate. Patients in our study returned to work after a median of 3 weeks, compared with 12 weeks after LRTI,^11^ and we observed a complication rate of 8%, markedly lower than that reported for LRTI.^10^ Considering these factors, we view CMC denervation as a joint-preserving first-line option after failed nonoperative treatment, offering patients a less invasive choice with the understanding that symptoms may recur and later trapeziectomy may be needed. This staged approach may be especially attractive in younger patients, who have a higher risk of reoperation after LRTI and for whom denervation can defer more invasive surgery.^44^ Although we currently use denervation in a staged manner, it may also be a reasonable direct alternative to trapeziectomy. However, further comparative studies are needed to establish its relative efficacy before it can be regarded as such.

Although the observed long-term pain outcome was durable, we observed substantial intersubject variability. To better understand the factors influencing these differences, we analyzed prognostic factors for postoperative pain scores. Male patients showed greater improvement than female patients, and those who underwent extended (PIN/AIN) denervation or had STT involvement experienced less pain relief.

PIN/AIN denervation is sometimes added to CMC joint denervation, primarily based on individual surgeon preference and theoretical rationale, as described by Giesen et al.^30^ Anatomical studies suggest that the PIN may innervate the first dorsal interosseous muscle and contribute to sensation in the first web space, referred to as the “Froment–Rauber” nerve. Furthermore, a possible distal connection between the PIN and the deep branch of the ulnar nerve supports its potential sensory role in the thumb CMC joint.^30^ To our knowledge, this is the first study to compare outcomes of standard versus extended denervation. We observed that extended denervation was associated with worse outcomes, possibly due to the additional dorsal incision and dissection required. However, this finding should be interpreted with caution, as most extended procedures were performed by a single surgeon who routinely applied this technique, and the observed difference may partly reflect variation in surgical indication rather than treatment effect. In addition, STT OA was more prevalent in the extended denervation group and independently associated with poorer outcomes. As denervation remains a relatively new technique, the variation in results between standard and extended approaches suggests there is room for further refinement. This highlights the need for further research to determine which specific nerve branches should be targeted to optimize symptom relief and surgical outcomes. Given the lack of additional pain relief, extended (PIN/AIN) denervation does not appear to offer meaningful added value over standard denervation in the treatment of CMC OA. Based on our findings, its routine use cannot currently be recommended.

Our analysis showed that STT OA is independently associated with poorer outcomes after CMC denervation, consistent with previous literature.^47^ Although we were unable to correlate exact Eaton–Glickel stages with outcomes due to incomplete reporting, our results suggest that CMC denervation may be less suitable in the presence of concomitant STT OA.

Our study has several limitations. First, we did not include a control group of patients undergoing trapeziectomy. However, we consider such a comparison inappropriate, as CMC denervation is typically indicated for a different patient population than trapeziectomy. Second, although some data were missing, analyses indicated they were missing completely at random. To minimize potential bias, we applied advanced statistical techniques, including linear mixed models and joint modeling. Finally, the inclusion of patients who underwent simultaneous procedures or presented with concurrent diagnoses may have influenced the results. Nonetheless, we believe this reflects routine clinical practice and therefore enhances the generalizability of our findings.

A key strength of this study is that it presents the largest cohort to date on CMC joint denervation, accompanied by long-term follow-up. The large sample size, multicenter design, and inclusion of multiple surgeons enhance the generalizability of the findings across diverse clinical settings. Moreover, this is the first study to examine prognostic factors associated with treatment outcomes after CMC denervation. Although our initial findings on prognostic factors are exploratory, further research involving larger cohorts is necessary to better determine which patient subgroups are most suitable candidates for this procedure.

## CONCLUSIONS

Joint denervation is an effective treatment for CMC OA, providing rapid recovery and a low complication rate. The majority of patients experienced sustained pain relief and improved hand function on long-term follow-up, with only 28% of thumbs undergoing a reoperation during the long-term follow-up. An extended (PIN/AIN) denervation involves an additional incision without yielding better pain outcomes; therefore, we do not recommend this additional procedure.

## DISCLOSURE

Dr. Eberlin is a consultant for AxoGen, Inc., Checkpoint Surgical, Inc., Integra Lifesciences Corporation, Tissium SA, Tulavi Therapeutics, Inc., BioCircuit Technologies, Inc., and Encoded Therapeutics, Inc. The remaining authors have no financial relationships or conflicts of interest to disclose. No financial support was received for the research, authorship, or publication of this article.

## ACKNOWLEDGMENTS

The authors thank the patients for completing questionnaires as part of their clinical care and for consenting to the anonymous use of their data, as well as the Hand-Wrist Study Group and the staff of Xpert Clinics and Equipe Zorgbedrijven for their contributions to the routine outcome measurements underlying this study.

## Supplementary Material



## APPENDIX

The members of the Hand-Wrist Study Group are as follows: R. Arjen M. Blomme, MD; Jeroen M. Smit, MD, PhD; Kennard Harmsen, MD; Gertjan Halbesma, MD; Guus M. Vermeulen, MD, PhD; Hans P. de Schipper, MD; Jeroen H. van Uchelen, MD, PhD; Oliver T. Zöphel, MD, PhD; J. Sebastiaan Souer, MD, PhD; Lisa Esteban Lopez, PT, CHT-NL; Alexandra Fink, PT, CHT-NL, MSc; Rob van Huis, PT, CHT-NL; Pierre-Yves A.A. Pennehouat, PT, CHT-NL; Karin Schoneveld, PT, CHT-NL, MSc; Grada R. Arends, OT, CHT-NL, MSc; Reinier Feitz, MD, PhD; Lisa Hoogendam, MSc; Steven E.R. Hovius, MD, PhD; Yara E. van Kooij, PT, CHT-NL, MSc; Jaimy E. Koopman, MD; Mark J.W. van der Oest, MD, PhD; Willemijn A. de Ridder, PT, CHT-NL, MSc; Ruud W. Selles, PhD; Liz-Tipper Sikking; Harm P. Slijper, PhD; Marloes H. P. ter Stege, MSc; Joris S. Teunissen, PhD; Robbert M. Wouters, PhD, PT, CHT-NL; Nina L. Loos, BSc; Nienke H.A. Mendelaar, MD; Lyse van Wijk, BSc; Ward R. Bijlsma, MD, PhD; Joost W Colaris, MD, PhD; Liron S. Duraku, MD, PhD; E.P.A. Brigitte van der Heijden, MD, PhD; Caroline A. Hundepool, MD, PhD; and J. Michiel Zuidam, MD, PhD.
