# Supplementary material for: Is Joint Denervation a Durable Treatment for Thumb Carpometacarpal Osteoarthritis? A Multicenter Long-Term Outcome Study
Source: Plast Reconstr Surg. 2026 Aug 26;158(3):40S–51S. doi: 10.1097/PRS.0000000000012905 (PMC13502385; doi:10.1097/PRS.0000000000012905)
Supplement: Supplementary file 2 [file prs-158-40s-s002.pdf]

Table, Supplementary Digital Content 2.

Table providing an overview of concurrent diagnoses and additional surgical procedures performed alongside CMC joint denervation.

Concurrent diagnosis	Number of patients (N = 21)
De Quervain's tenosynovitis	3
Osteoarthritis (OA) of the metacarpophalangeal (MCP) and distal interphalangeal (DIP) joints + tenosynovitis in ray 1 and 3	2
Carpal Tunnel Syndrome (CTS)	2
Dupuytren's nodules	2
Proximal interphalangeal (PIP) joint and DIP joint OA	2
Interphalangeal (IP) joint OA	1
Pisotriquetral OA	1
Radiocarpal OA	1
Scapholunate advanced collapse (SLAC) grade 3/4	1
Trigger thumb and De Quervain's tenosynovitis	1
DIP-3 joint OA bilaterally	1
MCP-1 instability	1
Radiocarpal and distal radioulnar OA	1
Scaphoid Nonunion Advanced Collapse (SNAC)/SLAC grade 4	1
Ulnar neuropathy	1

Concurrent surgery	Number of patients (N = 12)
Carpal tunnel release	2
De Quervain's release	2
Trigger finger release (TVR)	2
Wrist denervation indicated for radiocarpal osteoarthritis	2
Guyon canal release	1
IP arthrodesis	1
Pisiformectomy	1
De Quervain's release and TVR	1
