# Supplementary material for: Is Joint Denervation a Durable Treatment for Thumb Carpometacarpal Osteoarthritis? A Multicenter Long-Term Outcome Study
Source: Plast Reconstr Surg. 2026 Aug 26;158(3):40S–51S. doi: 10.1097/PRS.0000000000012905 (PMC13502385; doi:10.1097/PRS.0000000000012905)
Supplement: Supplementary file 3 [file prs-158-40s-s003.pdf]

Table, Supplementary Digital Content 3.

Table that compares the baseline demographic characteristics of patients undergoing standard versus extended denervation.

**Variable**	**Denervation (N=53)**	**Extended (PIN/AIN) denervation (N=23)**	**SMD**	**p-value**
Age (y), mean (SD)	57.7 (10.5)	55.0 (9.8)	0.268	0.293
Sex (female), N (%)	41 (77)	19 (83)	0.131	0.834
Dominant side treated, N (%)	21 (40)	12 (52)	0.254	0.446
Occupational intensity, N (%)			0.735	0.050
Unemployed	14 (26)	4 (17)		
Light physical labor	16 (30)	3 (13)		
Moderate physical labor	9 (17)	11 (48)		
Heavy physical labor	14 (26)	5 (22)		
Second opinion, N (%)	4 (8)	5 (22)	0.410	0.170
Concurrent diagnosis, N (%)	15 (28)	6 (26)	0.050	>0.999
Concurrent surgery, N (%)	8 (15)	4 (17)	0.062	>0.999
STT joint involvement, N (%)	9 (17)	7 (30)	0.320	0.310
VAS pain at rest score, mean (SD)	51 (24)	52 (23)	0.080	0.787
VAS pain during exertion score, mean (SD)	71 (20)	66 (25)	0.218	0.445
VAS function score, mean (SD)	44 (23)	49 (19)	0.206	0.582

N number of patients, *y* years, *SD* standard deviation, *VAS* Visual Analogue Scale, *PIN* posterior interosseous nerve, *AIN* anterior interosseous nerve, *STT* scaphotrapeziotrapezoid

SMD Standardized Mean Difference; around 0.2 indicates a small difference, around 0.5 a moderate difference, and around 0.8 a large difference.[35]
