# Supplementary material for: Is Joint Denervation a Durable Treatment for Thumb Carpometacarpal Osteoarthritis? A Multicenter Long-Term Outcome Study
Source: Plast Reconstr Surg. 2026 Aug 26;158(3):40S–51S. doi: 10.1097/PRS.0000000000012905 (PMC13502385; doi:10.1097/PRS.0000000000012905)
Supplement: Supplementary file 4 [file prs-158-40s-s004.pdf]

Table, Supplementary Digital Content 4.

Table that illustrates the non-responder analysis at intake.

**Variable**	**Non-responder (N=23)**	**Responder (N=53)**	**SMD**	**p-value**
Age (y), mean (SD)	56.0 (8.8)	57.3 (10.9)	0.123	0.638
Sex (female), N (%)	17 (74)	43 (81)	0.174	0.687
Dominant side treated, N (%)	9 (39)	24 (45)	0.125	0.806
Occupational intensity, N (%)			0.922	**0.011**
Unemployed	2 (9)	16 (30)		
Light physical labor	8 (35)	11 (21)		
Moderate physical labor	3 (13)	17 (32)		
Heavy physical labor	10 (44)	9 (17)		
Second opinion, N (%)	2 (9)	7 (13)	0.145	0.715
Concurrent diagnosis, N (%)	4 (17)	17 (32)	0.345	0.266
Concurrent surgery, N (%)	3 (13)	9 (17)	0.110	>0.999
Extended (PIN/AIN) denervation, N (%)	6 (26)	17 (32)	0.132	0.802
STT joint involvement, N (%)	5 (22)	11 (21)	0.024	>0.999

N number of patients, *y* years, *SD* standard deviation, *PIN* posterior interosseous nerve, *AIN* anterior interosseous nerve, *STT* scaphotrapeziotrapezoid
SMD Standardized Mean Difference; around 0.2 indicates a small difference, around 0.5 a moderate difference, and around 0.8 a large difference.[35]
