# Supplementary material for: Is Joint Denervation a Durable Treatment for Thumb Carpometacarpal Osteoarthritis? A Multicenter Long-Term Outcome Study
Source: Plast Reconstr Surg. 2026 Aug 26;158(3):40S–51S. doi: 10.1097/PRS.0000000000012905 (PMC13502385; doi:10.1097/PRS.0000000000012905)
Supplement: Supplementary file 5 [file prs-158-40s-s005.pdf]

Table, Supplementary Digital Content 5.

Table that illustrates the non-responder analysis at three-month follow-up.

**Variable**	**Non-responder (N=38)**	**Responder (N=38)**	**SMD**	**p-value**
Age (y), mean (SD)	55.8 (9.4)	58.0 (11.2)	0.204	0.376
Sex (female), N (%)	28 (74)	32 (84)	0.260	0.399
Dominant side treated, N (%)	16 (42)	17 (45)	0.053	>0.999
Occupational intensity, N (%)			0.813	**0.013**
Unemployed	6 (16)	12 (32)		
Light physical labor	12 (32)	7 (18)		
Moderate physical labor	6 (16)	14 (37)		
Heavy physical labor	14 (37)	5 (13)		
Second opinion, N (%)	4 (11)	5 (13)	0.082	>0.999
Concurrent diagnosis, N (%)	9 (24)	12 (32)	0.177	0.608
Concurrent surgery, N (%)	6 (16)	6 (16)	<0.001	>0.999
Extended (PIN/AIN) denervation, N (%)	11 (29)	12 (32)	0.057	>0.999
STT joint involvement, N (%)	10 (26)	6 (16)	0.260	0.399
Preoperative VAS pain at rest, mean (SD)	45 (24)	54 (23)	0.359	0.238
Preoperative VAS pain during exertion, mean (SD)	71 (20)	69 (23)	0.116	0.714
Preoperative VAS function, mean (SD)	39 (19)	48 (22)	0.450	0.247
6 weeks VAS pain at rest, mean (SD)	28 (7)	37 (22)	0.564	0.494
6 weeks VAS pain during exertion, mean (SD)	49 (17)	52 (24)	0.168	0.815
6 weeks VAS function, mean (SD)	60 (2)	61 (27)	0.039	0.963

N number of patients, *y* years, *SD* standard deviation, *VAS* visual analog score, *PIN* posterior interosseous nerve, *AIN* anterior interosseous nerve, *STT* scaphotrapeziotrapezoid
SMD Standardized Mean Difference; around 0.2 indicates a small difference, around 0.5 a moderate difference, and around 0.8 a large difference.[35]
