# Supplementary material for: Is Joint Denervation a Durable Treatment for Thumb Carpometacarpal Osteoarthritis? A Multicenter Long-Term Outcome Study
Source: Plast Reconstr Surg. 2026 Aug 26;158(3):40S–51S. doi: 10.1097/PRS.0000000000012905 (PMC13502385; doi:10.1097/PRS.0000000000012905)
Supplement: Supplementary file 6 [file prs-158-40s-s006.pdf]

Table, Supplementary Digital Content 6.

Table that illustrates the non-responder analysis at long-term follow-up.

**Variable**	**Non-responder (N=29)**	**Responder (N=47)**	**SMD**	**p-value**
Age (y), mean (SD)	55.6 (9.9)	57.7 (10.6)	0.207	0.387
Sex (female), N (%)	23 (79)	37 (79)	0.014	>0.999
Dominant side treated, N (%)	15 (52)	18 (38)	0.272	0.363
Occupational intensity, N (%)			0.297	0.671
Unemployed	6 (21)	12 (26)		
Light physical labor	8 (28)	11 (23)		
Moderate physical labor	6 (21)	14 (30)		
Heavy physical labor	9 (31)	10 (21)		
Second opinion, N (%)	5 (17)	4 (9)	0.263	0.290
Concurrent diagnosis, N (%)	8 (28)	13 (28)	0.002	0.608
Concurrent surgery, N (%)	5 (17)	7 (15)	0.002	>0.999
Extended (PIN/AIN) denervation, N (%)	7 (24)	16 (34)	0.219	0.512
STT joint involvement, N (%)	7 (24)	9 (19)	0.121	0.819
Preoperative VAS pain at rest, mean (SD)	53 (23)	50 (24)	0.090	0.758
Preoperative VAS pain during exertion, mean (SD)	75 (14)	67 (25)	0.428	0.168
Preoperative VAS function, mean (SD)	48 (19)	44 (24)	0.182	0.588
3 months VAS pain at rest, mean (SD)	29 (28)	39 (25)	0.368	0.268
3 months VAS pain during exertion, mean (SD)	48 (30)	51 (22)	0.122	0.706
3 months VAS function, mean (SD)	68 (25)	55 (27)	0.468	0.182

N number of patients, *y* years, *SD* standard deviation, *PIN* posterior interosseous nerve, *AIN* anterior interosseous nerve, *STT* scaphotrapeziotrapezoid

SMD Standardized Mean Difference; around 0.2 indicates a small difference, around 0.5 a moderate difference, and around 0.8 a large difference.[35]
