# Supplementary material for: Is Joint Denervation a Durable Treatment for Thumb Carpometacarpal Osteoarthritis? A Multicenter Long-Term Outcome Study
Source: Plast Reconstr Surg. 2026 Aug 26;158(3):40S–51S. doi: 10.1097/PRS.0000000000012905 (PMC13502385; doi:10.1097/PRS.0000000000012905)
Supplement: Supplementary file 7 [file prs-158-40s-s007.pdf]

Figure, Supplementary Digital Content 7.

Figure that illustrates patient satisfaction and willingness to undergo treatment again at 3 months and long-term follow-up.

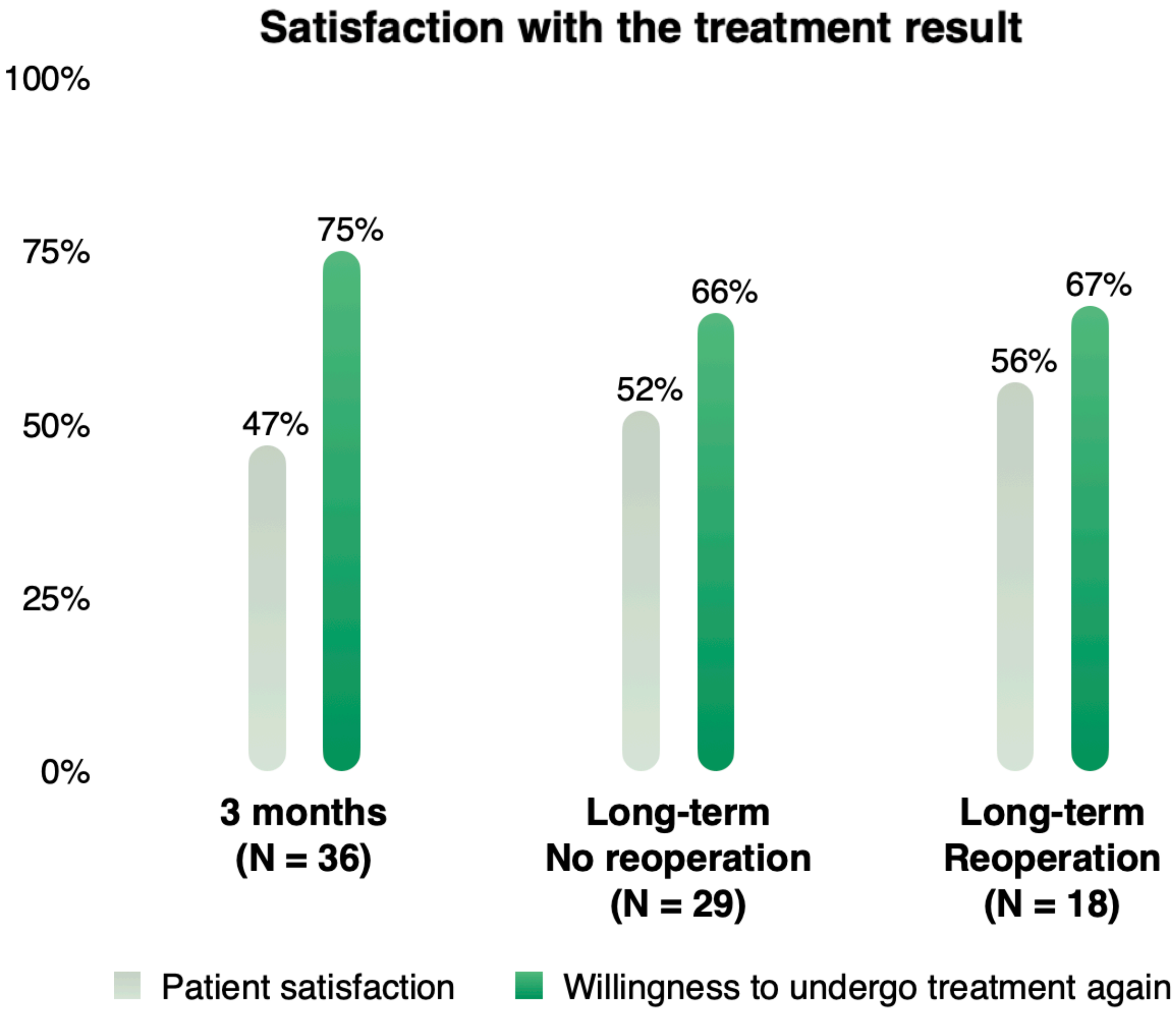

Patient satisfaction and willingness to undergo treatment again at 3 months and long-term follow-up, presented as percentages. For long-term outcomes, data are stratified by reoperation status. Notably, a significant proportion of patients who underwent reoperation remained satisfied with the denervation procedure and indicated they would choose to undergo it again.
